# Methodological framework for projecting the potential loss of intraspecific genetic diversity due to global climate change

**DOI:** 10.1186/1471-2148-12-224

**Published:** 2012-11-24

**Authors:** Markus Pfenninger, Miklós Bálint, Steffen U Pauls

**Affiliations:** 1 Biodiversity and Climate Research Centre (BiK-F) by Senckenberg Research Institut & Goethe University, Senckenberganlage 25, D-60325, Frankfurt/Main, Germany; 2 Molecular Biology Center, Babeş-Bolyai University, Str. Treboniu Laurian 42, 400271, Cluj, Romania

## Abstract

**Background:**

While research on the impact of global climate change (GCC) on ecosystems and species is flourishing, a fundamental component of biodiversity – molecular variation – has not yet received its due attention in such studies. Here we present a methodological framework for projecting the loss of intraspecific genetic diversity due to GCC.

**Methods:**

The framework consists of multiple steps that combines 1) hierarchical genetic clustering methods to define comparable units of inference, 2) species accumulation curves (SAC) to infer sampling completeness, and 3) species distribution modelling (SDM) to project the genetic diversity loss under GCC. We suggest procedures for existing data sets as well as specifically designed studies. We illustrate the approach with two worked examples from a land snail (*Trochulus villosus*) and a caddisfly (*Smicridea* (*S.*) *mucronata*).

**Results:**

Sampling completeness was diagnosed on the third coarsest haplotype clade level for *T. villosus* and the second coarsest for *S. mucronata*. For both species, a substantial species range loss was projected under the chosen climate scenario. However, despite substantial differences in data set quality concerning spatial sampling and sampling depth, no loss of haplotype clades due to GCC was predicted for either species.

**Conclusions:**

The suggested approach presents a feasible method to tap the rich resources of existing phylogeographic data sets and guide the design and analysis of studies explicitly designed to estimate the impact of GCC on a currently still neglected level of biodiversity.

## Background

Within the scientific community the evidence for a rapid and profound global climate change (GCC) is now widely accepted. Temperatures are predicted to rise by up to 4°C by 2100, as are severe changes in precipitation patterns [1]. It is therefore a major challenge to estimate and predict the consequences of GCC on biodiversity. Currently, the attention is focussed on predicting the effects on ecosystems and species. The third component of biodiversity as defined by the United Nations [2] – genetic diversity on the molecular level – has largely been neglected to date, despite being crucial for the maintenance of the evolutionary potential of species [3,4]. GCC will affect the extent and distribution of genetic diversity within species [5] as their ranges change [3]: Core areas of species ranges may become marginal with respect to their geographical [6-9] and/or ecological conditions [10]; metapopulation dynamics may change; areas that are newly colonised may undergo colonisation bottlenecks and/or the process of expansion itself may change the genetic composition of species e.g. by allele surfing [8,11-15], while populations in the trailing ends of shifting ranges may not move successfully but become extinct with all the intraspecific diversity they harboured threatened of being lost [16]. To gain an overview of the severity of the problem and to potentially implement mitigation strategies, accurate projections of the future distribution of intraspecific genetic variability are necessary.

While detailed predictions of the effect of random drift-based processes on genetic diversity are inherently difficult if not impossible to make, it should at least be possible to forecast the potential loss of genetic diversity associated with projected population extinctions or range shifts. However, to date, only few rigorous methodological frame works exists to perform such projections [17]. Several recent studies have implemented species distribution modelling (SDM) and future predictions of climatically suitable ranges to assess the effect of GCC on neutral genetic diversity assessment of non-model organisms [18-21]. However, three interrelated methodological issues that may compromise their information potential and statistical rigour are common to these studies: 1) the arbitrary choice of the assessed genetic diversity level, 2) the lacking assessment of how well genetic diversity was sampled, and 3) the choice of SDM projection scales relative to the sampling resolution. How these issues can compromise inferences on projected impacts of GCC on genetic diversity will be discussed in detail below. In a recent paper Jay et al. [22] proposed an approach aiming to assess the impacts of GCC on population structure from putative neutral markers across the genome based on their correlation with spatial and environmental data.

Following a different approach, we here present a methodological framework focussing on projecting the potential loss of neutral intraspecific genetic variation based on the projected extinction of populations resulting from GCC induced changes in species distribution ranges. It consists of 1) identifying an appropriate hierarchical level of genetic diversity for inference from a sample of individual genotypes or haplotypes by 2) assessing the completeness of sampling these hierarchical levels, 3) choosing the appropriate spatial scale for SDM modelling and finally 4) projecting the loss of currently occupied species range and the associated loss of genetic variation. The suggested approach combines existing, established methods in an innovative fashion. As we will show, there is no straightforward “one-strategy-fits-all” solution, but we offer suggestions how to optimize the GCC projections for individual species depending on the intended scope of the study and previous knowledge in an iterative process. In addition, the problems are different for studies explicitly designed for this purpose and the post-hoc analysis of existing data sets e.g. of phylogeographic studies. We will address the problems in turn, then suggest solutions and finally illustrate the approach in two worked examples.

## Methods

### Appropriate choice of the level of genetic variation for projections

In order to quantify a relative loss from a set of objects, we necessarily need to know how large the set initially was. For example, the statement that we lost half our money requires knowing how much we initially had. And, of course, we must have a clear definition of the set of objects we want to quantify and their value. To remain with the example, the extent of our economic disaster depends not only on the amount initially in our purse, but also on the values of the respective currency units lost.

Genetic variation is a very broad, fuzzy concept that may refer to very different scales within an individual, population or species. For example in a population genetic variation may relate to heritable phenotypic variation such as flower colour or body size, multilocus allozyme genotypes or to haplotypes made up of SNPs on a non-recombining DNA stretch. These different levels of genetic variation are not easily compared, even though they may all be traced back to their basis, i.e. to differences on the DNA and how these differences are combined in alleles and their combinations, at least in principle. The number of DNA variations and their potential combinations within a sexually reproducing species is almost infinitely large [23]. Basically the same is true for the cytoplasmic genomes in mitochondria and chloroplasts where there are possibly several thousands of unique haplotypes within a species. When faced with the task to quantify loss of genetic diversity due to GCC, it is therefore necessary for each study to first identify and explicitly define a level of genetic diversity that finds a meaningful balance between the practically unlimited finely scaled genetic variation within most species and the available resources to assess them. As we will show below, the appropriate scale or level of genetic variation for a planned assessment of genetic diversity can be determined individually for each study based on the sampling effort in terms of the number of genetic markers employed, sites or populations sampled and individuals screened.

Studies dealing with range wide neutral genetic diversity losses, however, are usually not interested in the fate of particular alleles or haplotypes (but see [22]) but rather in that of units with some evolutionary significance, i.e. non-random combinations of alleles and haplotypes that arose either by drift due to some degree of (geographical) isolation or local adaptation or both [24]. Such combinations may constitute every degree of evolutionary distinctness from random drift combinations over locally adapted populations to full-fledged cryptic species [25]. The molecular ecology literature offers ample evidence that these different diversity units are often also ecologically different as a consequence of their evolutionary divergence [e.g. [26-28]]. Such *evolutionary units, lineages, clades* or *clusters* (these terms will be used interchangeably hereafter) are usually successfully inferred using supposedly neutral genetic markers, like SNPs, AFLPs, microsatellites or mitochondrial sequences in animals, and chloroplast sequences in plants [24]. Evolutionary units can be defined on various *hierarchical levels*, depending on the level of genetic distinctness and on the resolution of the molecular marker systems applied. Often the former is a function of the latter. For example, a particular combination of SNPs along a stretch of non-recombining DNA makes up a haplotype. Depending on their ancestry, these haplotypes form monophyletic groups that can be joined with other such groups to higher level monophyletic groups. Likewise, multilocus genotypes may be most similar among individuals within families, a group of families forms a population distinguishable from other such populations by their genetic make-up and groups of populations within a species may be genetically more similar to each other than to populations of other such groups. In a study that sampled and genetically characterized individuals of a species each haplotype or genotype that is revealed represents an *instance* of each of the hierarchical levels it belongs to. Due to increasingly older shared common ancestry, each of those hierarchical levels thus consists of a decreasing, ever more inclusive number of genetic *entities* with increasingly higher position in the hierarchy. For example haplotypes that differ by a single base pair (bp) change along an analysed stretch of DNA can be nested into 1-step haplotype clades, and these 1- step clades can then be nested into 2-step haplotype clades etc. [29]. In such a hierarchy the genetic difference, i.e. the number of bp changes along the analysed stretch of DNA individuals than within clades, and the differences generally increase among individuals from lower to higher levels of hierarchy. The inference of such hierarchical levels can be based on a variety of different algorithms ranging from simple distance approaches to more complex algorithms that combine phylogenetic and coalescent models [30].

While we cannot hope to account for the fate of every single base pair substitution, it should equally not be the goal to assess only the coarsest hierarchical level of genetic variation in any given intraspecific study. Additionally, a sensible projection of the loss of genetic diversity is only possible with a robust estimation of the quantitative and spatial extend of currently existing diversity. We therefore argue that from a statistical point of view the highest resolution level of genetic variation where all genetic entities were or can be sampled at least once represents the level that was exhaustively sampled and is thus the appropriate level for projection. Identifying the level that is biologically most meaningful for any given species on the other hand remains the task of the researcher, is likely to vary from species to species, and depends on the marker system used.

### Assessing completeness of sampling

The sampling design for genetic studies should always be adequate for the intended purpose [31,32]. Independent of the hierarchical genetic level to be assessed, in studies wishing to project the impacts of GCC on genetic diversity, adequate sampling depends on 1) the degree of sampling completeness of the targeted level of genetic diversity at the individual sampling sites and 2) the adequate spatial coverage of the area of interest.

It is important to assess to which degree the samples from each individual sampling site contain all relevant genetic entities actually present at this site. Otherwise, the current extent of the spatial distribution of an entity may be seriously underestimated. This problem is similar to certain sampling issues in population genetics [33]. Here, the appropriate sampling effort in terms of the minimum number of individuals that should be sampled per site depends on the desired resolution of evolutionary units in question: while it might be acceptable to miss some rare genotypes/haplotypes that are threatened by drift or swamping in a freely reproducing population anyway, it may be detrimental to miss individuals of a rare sympatric cryptic species threatened by GCC. Basic probability calculations (see Additional file 1) show that if, for example, an entity that actually presents 10% of the population at a given sampling site shall be detected at least once with 95% certainty, then at least 29 instances (i.e. 14–15 individuals in case of a codominant diploid locus or 29 individuals for a haploid locus or for composite genotype cluster memberships, respectively) must be screened. If a unit of 5% frequency shall be detected with 99% probability, already 88 instances must be screened. An alternative method for inferring if all relevant units were sampled at a given site are individual based rarefaction curves. These, however, only work for sufficiently variable samples [34,35]. For existing data sets with already fixed sample sizes, the power of the analysis can be easily determined (see Additional file 1). For example, if 10 haplotypes were sampled in a population, the probability of having missed an entity with a true frequency of 10% is already 0.35.

**Figure 1:**
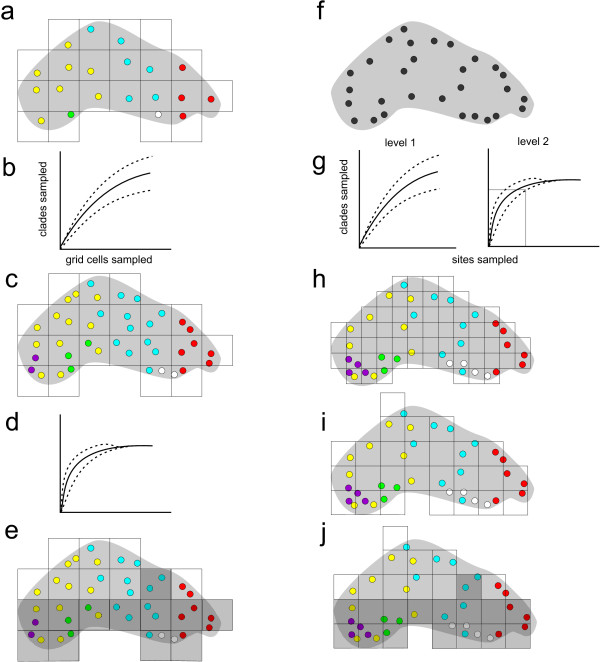
**Schematic procedure to estimate the loss of genetic diversity by global climate change. **The species range in this fictional example is shaded in grey. Sampling sites are shown as filled circles; the colour indicates the clade found at the respective sampling site. Grid cells for SDM modelling are indicated by squares, grid cells unsuitable in the future are shaded. It is assumed that the sampling sites have been comprehensively sampled (see text). **a-e)** Work flow for studies especially designed for the purpose of projecting genetic diversity losses under GCC. **a)** sampling required to achieve comprehensive coverage of desired spatial resolution, **b) **test whether sampling at desired clade level was complete, **c) **if necessary sample the grid cells again, **d) **test again for completeness, **e) **make projections. **f-i) **Work flow for an existing data set. **f) **existing, non-systematic sampling **g) **determine comprehensively sampled clade level and associated appropriate sampled number of grid cells for SDM, **h) **determine whether effective number of sampled grid cells is actually reached with given resolution and whether spatial sampling is appropriate, **i) **rescale grid if necessary, if repeated rescaling does not provide satisfactory results, sample additional sites, **j) **make projections.

If some knowledge on the genetic structure of the study organism is available, the desired inference level of intraspecific variation and the appropriate spatial sampling scale can be chosen *a priori* (Figure 1a). However, the adequacy of sampling can only be assessed empirically. To test whether and at which hierarchical level this goal was achieved, we propose using resampling techniques usually employed by field ecologists to determine the species richness of an area (Figure 1b). Sampling-site-based species accumulation curves (SAC) [34,35] assess whether the total sample is an accurate reflection of the total number of species in the area of interest. The rationale behind these methods is that the rate at which different sampled units or entities are added to the sample allows estimating their total richness [35]. It is assumed that the total number of species present was sampled if the resampling curve reaches saturation, in other words adding more samples does not increase the total number of species sampled. While species are usually the unit of inference for such analyses in ecological studies, genera or families i.e. monophlyletic units are also often used [35]. It is therefore straightforward to use subspecific entities like haplotypes or genotypes, as well as coarser resolution entities such as hierarchically nested haplotype clades or genotype cluster memberships for the same purpose.

For studies wishing to project genetic diversity losses we propose using SAC in one of two ways. If the level of inference was *a priori* determined, SACs can be used to assess whether the desired level was exhaustively sampled or if additional sampling is necessary (Figure 1b-d). For existing data sets, where additional sampling is often not feasible, SACs can be used at increasingly higher hierarchical levels of genetic variation. This is a straightforward and statistically sound approach to determine the appropriate level of inference, i.e. the lowest hierarchical level where sampling saturation is reached (Figure 1f-g).

### Assessing adequacy of spatial sampling

If the spatial distribution of the sampling sites in existing studies is sufficiently unbalanced, situations can arise where the SAC analysis indicates sampling saturation, yet clades were not sampled because they are geographically restricted to unsampled parts of the species range. It is of course also possible to miss higher order clades that are restricted to very small areas or even single populations [36] despite applying a fine-scale sampling design across a species range. However, such spatially very restricted clades at the level where sampling saturation was already diagnosed are likely the exception, because to reach saturation in SAC, the entities under scrutiny must necessarily occur in more than a single sampling site and thus have a certain minimal spatial range [34].

We suggest that the *potential dispersal range size* of the sampled clades may be used to assess whether the spatial sampling site distribution adequately covers the species range. We define the *potential dispersal range size* of a clade as the area of the circle whose perimeter goes through the furthest spaced sampling sites where the clade was found. The rationale behind this quantity is that the clade has spread at least this far during its existence. Calculating the potential dispersal range size of the least widely distributed clade in terms of occupied grid cells used for species distribution modelling (see below) should thus give a conservative expectation of the distribution range of a clade at the given hierarchical level. If one or more spatially coherent unsampled areas of this or larger size exists within the species range, sampling these areas should be considered.

### Choosing the appropriate scale of spatial inference for genetic diversity loss prediction

The next issue we address is the spatial scale at which projections of genetic diversity loss can be reasonably made. Please note that this is not a discussion of the accuracy or methodology of SDM modelling, which depends on the spatial accuracy of the species occurrence data used (see [37] for a recent review) but solely on the appropriate spatial grain of such projections. Note that the species occurrence data need not to be identical to the sites used for diversity loss predictions. There is usually a discrepancy between the spatial scale at which statistical climate niche modelling is performed (e.g. grids of 10, 2.5 or 0.5 arc-minutes for the popular WorldClim data layers) and the spatial scale of genetic sampling for phylogeographic studies (usually several tens or hundreds of kilometres between sampling sites). Such a mismatch of spatial scales and coverage may insinuate an accuracy of the genetic diversity loss prediction that is not warranted by the spatial coverage of the genetic data [38].

Because of the necessity to interpolate the spatial distribution of the genetic lineages, we may flag an entity as prone to extinction although it actually also occurs in an unthreatened, but not sampled site of an otherwise sampled area. Given that we are investigating the fate of genetic diversity at a level where sampling saturation was reached, such an approach is conservative in the sense that it will not completely miss a potentially threatened entity of interest, but we may overestimate the degree of extinction threat.

The choice of an appropriate sampling grid size should be guided by the empirical distribution of genetic variability in the focal species and the desired spatial resolution of the prediction. While it is obvious that the entire area of interest (the species’ range, a certain country or geographic region) should be adequately covered, it is less clear at the beginning of a study at which spatial resolution the sampling should be performed, i.e. which distance between sampling sites in adequate.

The density of the sampling is in principle only limited by the population density of the focal species, and in practice by the available resources. Therefore, the minimum number of spatial grid cells to sample should assure that saturation at the desired hierarchical level was reached. An initial hint of the appropriate sampling resolution may be gained from *a priori* knowledge of the home ranges, genetic population structure or the extent of spatial autocorrelation of the appropriate clade level of the focal organism. However, the complex interplay of spatially varying processes like life-history, demography, and dispersal but also contingent factors like population history, landscape features and natural selection will usually prevent any *a priori* predictability of the spatial distribution of genetic variability. It is thus likely that the sampling density needs to be adjusted in an iterative process of sampling successively more sites within the grids until saturation at the desired resolution is reached (Figure 1c,d).

The situation is different if one wants to make use of the large body of phylogeographic data already available as recently proposed [19,39]. Here, the grid size for spatial prediction needs to be reasonably adjusted to the existing sampling scheme. Sampling for phylogeographic studies is often not performed in a systematic fashion; i.e. some areas are sampled more densely than others so that regardless of grid size, some cells of the area of interest will usually remain empty unless there are far fewer grid cells than sites sampled (Figure 1h). Since the size of the area of interest is fixed, the task is to determine the number of grid cell resolution in a way to 1) maximizes the spatial resolution, and 2) still assure exhaustive sampling on the level of interest. We argue that the number of grid cells can be reasonably increased up to the point where a maximal number of randomly drawn grid cells still yields 95% of the genetic entities with 95% certainty (or any other predefined coverage thresholds). This number (N_cell_) can be determined as

(1) $$N_{\text{cell}}=N_{\text{sam}}+\left(N_{\text{sam}}–N_{\text{min}}\right)$$

whereby N_sam_ denotes the number of actually sampled grid cells and N_min_ the minimum number of sampled grid cells necessary to reach the desired coverage. The latter can be determined with the resampling technique described above but with sampled grid cells as the unit and not the individual sampling sites. The maximum number of grid cells sampled cannot exceed the number of sites sampled. The value derived from the latter is thus a good starting point from which the optimal cell size can be empirically determined in an iterative process by successively decreasing N_cell_ and repooling the data according to their distribution in the grid cells. Such a rescaling towards a coarser grain size does not influence the SDM performance [40]. The expectation is that the more evenly spaced the sample sites are, the higher the spatial resolution that can be obtained, because less sample sites will fall into the same grid cell. To increase spatial resolution, the grid position should maximize the number of occupied grid cells (Figure 1h-i). However, the number of sampled grid cells should not fall below 30 to achieve reliable SDMs [38].

### Worked example

#### Data sets

We could not find a published data set that comprehensively met the criteria outlined above in terms of spatial coverage and sampling depth. Therefore, we used two published data sets that are representative for very well and more poorly sampled phylogeographic data sets: one deeply and methodologically sampled phylogeography data set on a land snail species [41] and one more shallowly but geographically comprehensively sampled DNA barcoding data set on a caddisfly species [42].

*Trochulus villosus* is a land snail from the Hygromiidae family currently distributed at altitudes between 400 and 2300 metres in a relatively confined area that includes Switzerland North of the main ridge of the Alps and adjacent areas in France, Germany and Austria [43]. The species survived the last glaciations in two isolated refugia in ice-free refugia in the Jura mountains close to the glacier margins [41]. From there, recolonisation of the present range took place. Climate change is expected to alter not only latitudinal but also altitudinal distributions of the snail [41]. It is therefore interesting to see whether there are evolutionary units that occur on the upper or lower altitudinal distribution margins and that are thus potentially particularly threatened by a warming climate, because the snails as proverbially poor active dispersers may not be able to track their shifting climate niche in time. The sampling for the phylogeographic study was performed in a systematic fashion by sampling sites approximately every 20 km within the known range.

**Figure 2:**
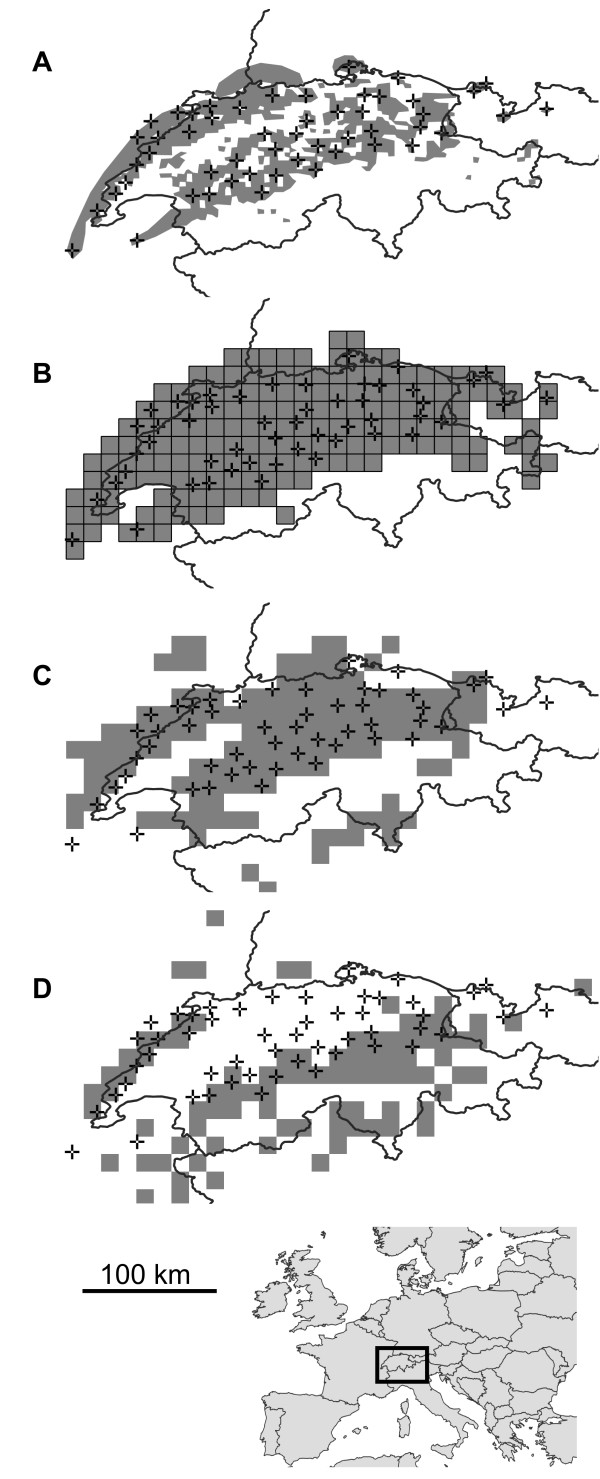
**Potential present and future distributions of T. villosus. **Species distribution modelling of *T. villosus* across its range in Switzerland and adjacent countries. The present range **(A) **and projections of suitable areas **(B-D) **are shaded grey; sampled populations are represented by crosses ( + ). **A) **the actual present distribution inferred from literature and sampling, **B) **the present distribution projected on the chosen spatial grid scale of SDM inference, **C) **the present distribution modelled from present climate data, **D) **the projected distribution in 2080 according to the IPCC A2 emission scenario.

In total, 455 individuals were sampled from 52 sampling sites (Figure 2). Sampling depth per site ranged between 4 and 10 individuals with a mean (s.d.) of 8.75 (1.49). This means that on average entities comprising about a quarter (0.27) of the total population have been detected with 95% probability at least once. The present analysis was based on the COI data set consisting of an alignment of 556 bp length (GenBank accession numbers EU025399 – EU025550). The data set contained 107 unique COI haplotypes. Each site harboured between one and six different haplotypes with a mean of 3.50 (1.48).

**Figure 3:**
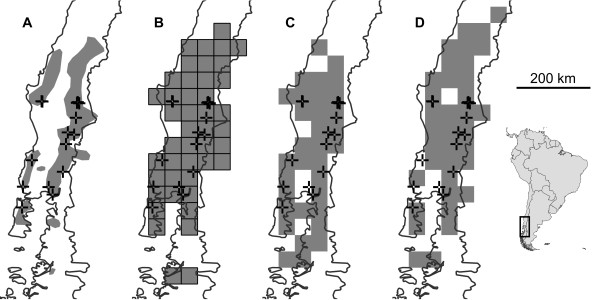
**Potential present and future distributions of S. mucronata. **Species distribution modelling of *S. mucronata *across its range in Chile and Argentina. The present range **(A) **and projections of suitable areas **(B-D) **are shaded grey; sampled populations are represented by crosses ( + ). **A) **The current distribution inferred from literature and sampling, **B) **The present distribution projected on the chosen spatial grid scale of SDM inference, **C) **the present distribution modelled from present climate data, **D)** the projected distribution in 2080 according to the IPCC A2 emission scenario.

*Smicridea* (*Smicridea*) *mucronata* Flint 1989 is a net-spinning caddisfly of the family Hydropsychidae distributed from close to sea level to above 1000 m a.s.l. in the coastal ranges and Andes of central Chile [42,44] (Figure 3). A DNA barcoding study of genus *Smicridea* (*S.*) *S. mucronata* revealed shallow but distinct clades within the coastal ranges and the Andes, though clades were shared among regions [42]. At present the species is restricted to cool swift running streams, and has been observed as a strong flier and swarming species (Pauls, personal observation). Because the species occurs in relatively high mountain ranges at moderate altitudes and is presumably a better disperser than the land snail it is likely that it could track its climatic niche within the ranges it currently inhabits under GCC conditions more readily than *T. villosus*.

The sampling comprises 97 individuals from 17 sites; one individual from the original data set was removed for our analysis because 2 base pairs were unresolved. In [42] the sampling was designed to cover every isolated mountain region in the Coastal Ranges or region of the Andes from which the species is known, but for a DNA taxonomy study and not explicitly for phylogeographic inference. Sampling depth was thus shallower and ranged from 1 to 11 individuals, with a mean (s.d.) of 5.71 (3.48). Therefore, entities must have been on average present in a frequency above 40% to ensure that they have been sampled at least once with 95% probability. The present analysis is based on an alignment of 658bp length (GenBank Accession numbers: HM065285-HM065379, HM065381, HM065382). The data set contained 23 unique COI haplotypes. Each site harboured between one and four haplotypes with a mean of 2.13 (1.02).

#### Hierarchical clustering

**Figure 4:**
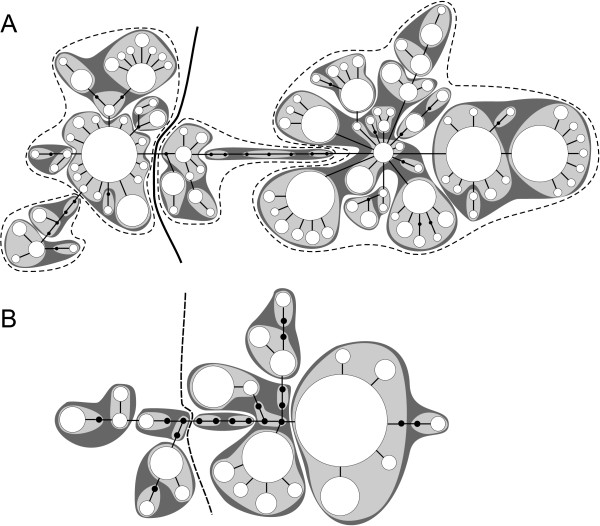
**Nested statistical parsimony cladograms of COI haplotypes. **White circles represent haplotypes. Their diameter is proportional to the number of individuals carrying that haplotype. Unsampled or extinct haplotypes are depicted as small black circles. Connection lines between haplotypes correspond to a single base substitution. Haplotypes united in 1-step clades are highlighted by light grey, two step clades by dark grey areas. The 3-step clades are separated by hatched lines, 4-step clades by solid lines. **A) **The 4-step nested cladogram of *T. villosus *and **B) **the 3-step cladogram of *S. mucronata*.

To infer higher level evolutionary units we used a nested clade design that successively links the closest genetic variants in a species [31]. The highest resolution of genetic variation we considered in the mtDNA sequence data was at the level of unique haplotypes. The phylogenetic relation between the haplotypes was inferred with statistical parsimony in the software TCS [45] and drawn as a network (Figure 4). Circular connections were broken according to the rules detailed in [46]. The haplotypes were nested according to the rules given in [29].

#### Clade accumulation curves

The clade/site matrices were used to calculate clade accumulation curves with 95% confidence intervals for each clade level in EstimateS vers. 8.20 [47]. We used default settings with 1000 randomizations for each run.

#### Clade dispersal range size estimation

The distance between the sampling sites furthest apart of the clade with the smallest spatial distribution was used to calculate the clade dispersal range size as a circle with this distance as diameter.

#### Species range estimation and climate niche modelling

The resolution of climatic layers used for the SDMs was estimated by dividing the distribution area size with the previously calculated number of grid cells. As argued above, the number of grid cells should yield at least 95% of the genetic entities with 95% certainty.

Bioclimatic layers with a resolution of 10 arc-minutes for the present conditions were downloaded from the public WorldClim database (http://www.worldclim.org, [48]). Future projections were based on the 4^th^ assessment of the Intergovernmental Panel for Climate Change for 2080 A2a CO_2_ emission scenario [1]. These were downloaded from the CIAT GCM downscaled data portal (http://ccafs-climate.org/,[49]). The bioclimatic layers were upscaled to the calculated resolutions in GRASS.

The potential present distribution of both species was computed with a maximum entropy approach [50] in Maxent v. 3.3.3 [51]. A general description of the method can be found in [52]. The models were trained on 75% of the locality information, and were tested on the remaining 25%. The predictions were cross-validated in 10 runs. Model performance was evaluated with the area under curve statistics (AUC, [53]). The values of the distribution probability maps were transformed into presence-absence values by applying a logistic threshold which maximizes the sensitivity and specificity of the projections.

## Results

### Hierarchical nesting

In case of *T. villosus,* the nesting comprised 5 levels, including the haplotypes (Figure 4A). For each clade level, a clade/site matrix was constructed. In *T. villosus* there were 107 haplotypes, 37 1-step clades, fourteen 2-step clades, four 3-step clades, and two 4-step clades (Figure 4A). In *S. mucronata* there were 23 haplotypes, eleven 1-step clades, six 2-step clades, and two 3-step clades (Figure 4B).

### Clade accumulation curves

**Figure 5:**
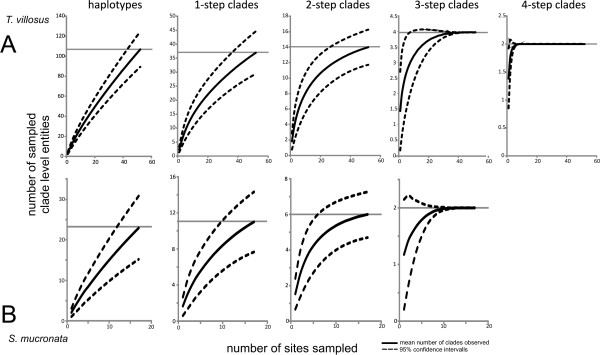
**Clade accumulation curves. **Clade accumulation curves for **A) ***T. villosus *and **B) ***S. mucronata *obtained by 1000 resamples. For each clade level, the observed number of clades is indicated by a horizontal line. The average number of sampled clades per site sampled is represented by a solid curve, the respective upper and lower 95% confidence limits by hatched lines. Saturation as indicated by the convergence of the confidence lines on the observed values is reached for both species on the 3-step level.

In *T. villosus* clade level saturation was reached at the 3-step clade level (Figure 5A). Analysis of the accumulation curve indicated that sampling of 30 sites will yield 95% of the total number of clades with 95% probability. This indicates that following Formula 1 dividing the species range of *T. villosus* into 52 + 52 – 30 = 74 grid cells is an appropriate starting value given the sampling. In *S. mucronata*, saturation was reached only at the highest clade level (3-step clade level, Figure 5B). In this case, sampling of 8 sites will be sufficient to sample 95% of the clades with 95% probability. So dividing the species range initially into 24 cells is appropriate for the data set.

### Species range and (re)scaling of SDM grid cells

The distribution area of *T. villosus* was calculated from the Centre Suisse de Cartographie de la Faune (CSCF) database [54], which contains locality information about the Swiss fauna at a resolution of 5 × 5 km^2^. This was completed with distribution estimates for France, Germany and Austria on the basis of [55] and the experience from the sampling effort for the phylogeographic study (Depràz, personal communication), and resulted in a distribution area of 19,782 km^2^. The distribution area of *S. mucronata* estimated from [42,44] was 62,407 km^2^. Area estimates were calculated in GRASS (GRASS Development Team 2011).

The initial calculation resulted in 10.7 arc-minute grid cells (equivalent of 267 km^2^ at 47 degrees latitude) for the systematically sampled *T. villosus.* The area of grid cells was 30.8 arc-minutes for *S. mucronata* (equivalent of 2496 km^2^ at 40 degrees latitude). This is under the assumption that sampling was performed in a way that each grid cell harbours at maximum a single site. However, when plotting the sampling localities over the upscaled bioclimatic layers, it became clear that the effective number of sampled grid cells was lower than the calculated thresholds. In the case of *T. villosus* the number of informative grid cells was 71, because three pairs of the 74 sampling localities fell into the same grid cell. In the case of the less densely sampled *S. mucronata* 4 of the 16 sampling localities fell into a grid cell with one or more other sampling sites, so the species’ range should be divided into 15 grid cells instead of 24. We therefore downscaled the 10 arc-minute grid cells once again. The final area of grid cells were only slightly larger (10.9 arc-minutes, 278 km^2^) for *T. villosus* (Figure 2)*,* but considerably different (39.8 arc-minutes, 4160 km^2^) for *S. mucronata* (Figure 3).

### Clade dispersal range size and spatial sampling adequacy

The sites furthest apart harbouring individuals from the least widely distributed clade on the 3-step level were 96 km apart in *T. villosus*. This corresponds to a clade dispersal range size of 7216 km^2^ or 26 grid cells at the chosen inference grid cell size of 278 km^2^. The largest patch of spatially coherent, unsampled grid cells was about 17 grid cells. The species range was thus adequately covered at the chosen inference level. The same was true for *S. mucronata*, where the calculated clade dispersal range size (118,628 km^2^) was larger than the entire distribution range (see above).

### Clade loss under climate change

SDM modelling suggested that under the chosen GCC scenario the suitable species range would be reduced by almost 30% for *T. villosus* (Figure 2B) and about 33% for *S. mucronata* (Figure 3B). In *T. villosus* 21 populations were sampled in grid cells where the suitable climate niche is threatened to vanish. On the relevant 3-step clade level, none of the four clades identified would completely vanish. However, one of these clades would remain only in a single grid cell. In *S. mucronata* 3 sampled populations are predicted to vanish. None of the two 3-step clades occurred exclusively at these sampling sites.

## Discussion

The methodological framework outlined here presents one of the first attempts to outline a statistically sound approach for estimating the effect of GCC on intraspecific genetic diversity as a consequence of projected range losses. As shown above, already sampling all haplotypes or alleles present at a single sampling site can be quite demanding in terms of individuals that need to be screened in order to assure sampling completeness. Sampling at this depth and level of detail over entire species ranges greatly exceeds current standards for thorough phylogeographic studies, and is most probably not possible for non-model organisms with current methodologies. On the other hand using thoroughly sampled phylogeographic studies can be a good starting point for studies assessing GCC on population genetic diversity as shown with the worked *Trochulus* example. Additional studies with other taxa will have to show whether the pattern of rather limited loss of genetic diversity projected here is typical.

Previous studies with similar aims lacked an explicit assessment on whether the chosen level of genetic diversity was completely sampled. These studies have suggested a much more severe loss of intraspecific genetic diversity, even at the level of independent evolutionary lineages [19], more or less analogous to the higher level clades we defined here. In situations where projections were made for haplotypes (e.g. [19]) or genotypes [20,21], it is likely that sampling saturation was not reached for the individual sampling sites nor for the entire species range (see Figure 5 for comparison). Thus, any quantitative and qualitative calculation of potential losses may be meaningless because the base line to which the projected losses were compared were only the haplotypes sampled and not all actually existing haplotypes. The use of haplotypes for projecting GCC effects pertains also to the second issue mentioned above, the choice of the genetic diversity level relevant for evolutionary or adaptive processes. The number of haplotypes or alleles that can be found in a study depends on the particular marker chosen as well as on the number of base pairs screened. Using a locus with different apparent mutation rate or even fragments of varying length of the same marker can yield more or less haplotypes/alleles and thus affect quantitative results. On the other hand, the use of higher order clades or evolutionary lineages is more or less independent of the marker(s) used as these can be expected to converge on higher evolutionary unit levels [56-58]. We argue that making GCC inferences on higher clade levels or the level of evolutionary independent lineages is reasonable, less likely to be flawed from insufficient sampling, and potentially more relevant from an evolutionary point of view. As an alternative to defining the biologically significant higher clade levels, the barcode gap has the potential to delimit units for such purposes but varies strongly among different taxa e.g. [42]. The GMYC species concept may also provide a tool that allows comparative studies [30], but here again the delimitation of units differs strongly among taxa e.g. [19]. All of the above approaches however are limited to defining evolutionary units based on haplotypes of a single gene region. Hierarchical genotype clustering methods (e.g. [58-60]) can be employed to incorporate multi-locus sequence data on the one hand, and multilocus genotypic data based on microsatellites, SNPs, AFLPs, or allozymes on the other hand. Currently, quantitative comparisons of the projected loss of molecular biodiversity among different species will remain difficult lacking a standard to compare evolutionary relevant units below taxonomic rank.

Separate evolutionary entities may have different responses to changing environmental conditions and therefore it could be useful to model their GCC-responses separately [61,62]. However, for a technical and a conceptual reason, we decided not to do so in the present case. The technical reason is that reliable SDM may not be possible for each separate evolutionary unit at higher resolution clades, because they may not be sampled sufficiently often (~30 sites per evolutionary unit [38]). This is e.g. the case both of the worked examples including the high resolution sampling performed on *T. villosus*.

The second, conceptual reason is that modelling the lineages separately requires the assumption that indeed the inferred lineages react as a unit to GCC and not only genes responsible for their presumed local adaptation (see respective discussion above). However, the resulting niche estimate is always a modelling-technique-dependent, more or less additive composite of the spatial distribution of all underlying entities, encompassing the respective niches of these subunits [63]. Using such a composite estimate would be critical if the goal was to project the direction and extent of range shifts for each of the subunits separately (which is not our focus). However, due to the additive nature of the niche estimates, the prediction which of current populations from the entire species range will be lost is uncritical. In other studies that apply units that are explicitly defined through a strong degree of isolation, e.g. GMYC species, modelling each unit separately would be appropriate and advisable.

It is recommended that the spatial scale of SDM should be consistent with the information content of the data [64]. This also applies in the present case. Scaling the grid size according to the effectively sampled number of grid cells is therefore useful, even if only for the assessment of spatial sampling adequacy.

It is well known that the quality of the projection depends strongly on the quality of the SDM, with all the known issues of e.g. microrefugia below SDM resolution where the genetic variation is at least partially preserved and local adaptation to the new conditions is possible. These problems and pitfalls also pertain to the forecasting of intraspecific genetic diversity, but we will not discuss them here, because they have been discussed at length elsewhere [64].

Predicting the fate of intraspecific genetic variation has additional issues that should be kept in mind when interpreting the results. For example, GCC may alter dispersal behaviour and in some cases provoke increased dispersal [65], which may results in a shift of intraspecific variation compared to present day distributions without a change to the actual distribution range [66]. Individuals from clades that were flagged as threatened may thus disperse to suitable areas if climatic pressure rises, preserving the genetic variation they carry. Conversely, generally increased dispersal may lead to swamping of locally adapted populations from more abundant populations [67]. As a variation to this scenario, the shift of selective regimes associated with a changing climate may favour selection driven gene-flow of respective functional alleles despite the climate driven loss of their populations of origin and the neutral variation associated with those lost populations. The projections gained from the proposed approach therefore present severe or even worst-case scenarios, which is not necessarily a drawback in nature conservation and follows the precautionary principle [68].

To demonstrate the performance of the proposed approach, we deliberately chose data sets that differ considerably in number of sampling sites, sampling depth at the sample sites and the spatial sampling site distribution (Figures 2 and 3). In both worked examples, no complete loss of mitochondrial haplotype lineages at the chosen level was predicted under the chosen GCC scenario, despite significant projected losses of suitable species range. However, for *S. mucronata* it was only possible to make inferences on the highest clade level, while in *T. villosus*, the second most coarse clade level could be used (Figure 5). This is in part due to the higher resolution in terms of number of sampling sites and individuals which likely led *per se* to a higher number of haplotypes compared to the shallower sampled *S mucronata* data set. Partly, however, the different resolution might also be explained by the different biology as well: the low dispersal capacity of land snails usually leads to stronger population differentiation than can be generally expected from flying insects. However, studies of winged aquatic insects have shown species-specific patterns of genetic population structure and population differentiation that suggest dispersal capacity varies dramatically even among ecological similar or closely related species [28,69,70], which is also true for land snails e.g. [71]. In *T. villosus*, the chosen 3-step clade level is already below the divergence level marking the two major glacial refugia and thus some potentially biologically relevant units (but see [41]). Whether the 3-step clades represent a Pleistocene substructure and to which extent this still has biological significance is not known. Whether the inferred 3-step clades in *S. mucronata* also mark a phylogeographic structure is unknown, because the data set was deemed unsuitable for such an analysis. However, the large geographic overlap and co-occurrence of only slightly disjunct clades at the same sites argues against a deeper biological significance of the inference clade level. In other species of *Smicridea* population structure and population differentiation are more pronounced, even at smaller geographic scales [42,70]. In *Trochulus*, highly divergent and reproductively isolated lineages may be restricted to single valleys [36,72,73]. In these species, losses of regional haplotypes or clades may thus have more direct biological significance.

The higher spatial coverage and the sampling depth of the *T. villosus* data set allowed for a higher prediction accuracy, because it was possible to find rarer occurrences. For example, the inference that none of the 3-step clades are threatened by extinction is based on two occurrences at a single sampling site in this species. The number of sites sampled and their spatial distribution also determined the spatial resolution for SDM. The projection grain in *T. villosus* was both absolutely (10.9 vs. 39.8 arc minutes) and relatively (1% vs. 7% of respective species range size) finer than in *S. mucronata*. The more thoroughly sampled *T. villosus* example thus gives much more confidence in the validity of the achieved results.

However, despite the substantial quantitative differences in the data sets, the presented data is a conservative prediction in the sense that we have, inherently to the approach, rather under- than overestimated the spatial distribution of the respective clades. Intraspecific genetic diversity is thus probably even less threatened than suggested here. These inferences may well differ under different climate scenarios, but the aim here was rather to illustrate the approach than to exhaustively analyse the data.

## Conclusions

The presented approach presents a feasible, sound methodological framework to 1) tap the rich resources of existing phylogeographic studies and 2) guide the design and analysis of studies explicitly aimed to estimate the impact of GCC on a currently largely neglected level of biodiversity.

## Competing interests

The authors declare to have no competing interests.

## Authors’ contributions

MP had the conceptual idea, MP and SP collected and analysed the data, MB performed the SDM modelling, MP drafted the manuscript and all authors contributed to writing the manuscript. All authors read and approved the final manuscript.

## Supplementary Material

Additional file 1 Determining the probability of having sampled an entity with a given frequency at least once. Click here for file
