# Supplementary material for: Methodological framework for projecting the potential loss of intraspecific genetic diversity due to global climate change
Source: BMC Evol Biol. 2012 Nov 24;12:224. doi: 10.1186/1471-2148-12-224 (PMC3538604; doi:10.1186/1471-2148-12-224)

Additional file 1

Determining the probability of having sampled an entity with a given frequency at least once.

As we want to determine whether a certain entity is present or not at a given site (as opposed to gain an estimate of its true proportion in the population), we need to sample it only once. If we assume that the population from which we sample is infinitely large, then the probability *p* not to randomly draw an entity of the true frequency x is 1-x for each independent draw. If we draw *n* times, the probability not to have drawn a single instance of the entity is $p = (1-x)^n$, or, inversely, the probability for having drawn the entity at least once is $1-p = (1-x)^n$. Thus, already for quite abundant entities it takes a substantial number of samples to encounter them at least once with high probability. For fixed sample sizes, the calculations can be used accordingly to infer the power of the analysis.

Additional file 1 Figure 1. Relation between sample size and probability of having sampled an entity of a certain frequency (coloured lines) at least once.

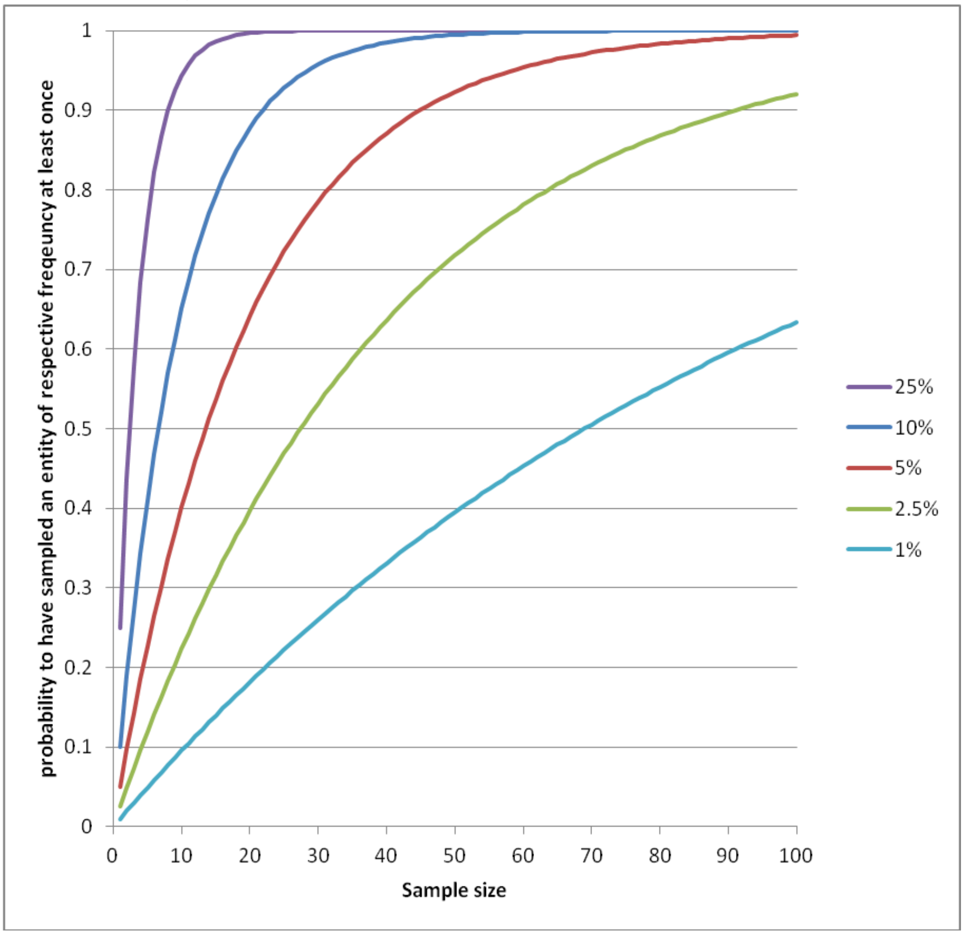

Supplement: Additional file 1 — Determining the probability of having sampled an entity with a given frequency at least once. [file 1471-2148-12-224-S1.pdf]
